# Tumour-derived CSF2/granulocyte macrophage colony stimulating factor controls myeloid cell accumulation and progression of gliomas

**DOI:** 10.1038/s41416-020-0862-2

**Published:** 2020-05-11

**Authors:** Malgorzata Sielska, Piotr Przanowski, Maria Pasierbińska, Kamil Wojnicki, Katarzyna Poleszak, Bartosz Wojtas, Dominika Grzeganek, Aleksandra Ellert-Miklaszewska, Min-Chi Ku, Helmut Kettenmann, Bozena Kaminska

**Affiliations:** 1 Laboratory of Molecular Neurobiology, Nencki Institute of Experimental Biology, Warsaw, Poland; 2 Max Delbruck Center, Molecular Neurosciences, Berlin-Buch, Germany

**Keywords:** Cancer microenvironment, Innate immune cells

## Abstract

**Background:**

Malignant tumours release factors, which attract myeloid cells and induce their polarisation to pro-invasive, immunosuppressive phenotypes. Brain-resident microglia and peripheral macrophages accumulate in the tumour microenvironment of glioblastoma (GBM) and induce immunosuppression fostering tumour progression. Macrophage colony stimulating factors (CSFs) control the recruitment of myeloid cells during peripheral cancer progression, but it is disputable, which CSFs drive their accumulation in gliomas.

**Methods:**

The expression of *CSF2* (encoding granulocyte-macrophage colony stimulating factor) was determined in TCGA datasets and five human glioma cell lines. Effects of stable *CSF2* knockdown in glioma cells or neutralising CSF2 or receptor CSF2Rα antibodies on glioma invasion were tested in vitro and in vivo.

**Results:**

CSF2 knockdown or blockade of its signalling reduced microglia-dependent glioma invasion in microglia-glioma co-cultures. CSF2-deficient human glioma cells encapsulated in cell-impermeable hollow fibres and transplanted to mouse brains, failed to attract microglia, but stimulated astrocyte recruitment. CSF2-depleted gliomas were smaller, attracted less microglia and macrophages, and provided survival benefit in tumour-bearing mice. Apoptotic microglia/macrophages were detected in CSF2-depleted tumours.

**Conclusions:**

*CSF2* is overexpressed in a subset of mesenchymal GBMs in association with high immune gene expression. Tumour-derived CSF2 attracts, supports survival and induces pro-tumorigenic polarisation of microglia and macrophages.

## Background

Glioblastoma (GBM) is a diffusive and highly malignant brain tumour, which is heavily infiltrated with brain-resident microglia, peripheral macrophages, granulocytes, and myeloid-derived suppressive cells (reviewed by refs. ^1–3^). Tumour-derived molecules act as attractants and functionally polarise infiltrating cells, so they promote extracellular matrix remodelling, support tumour invasion and angiogenesis, and contribute to local and systemic immunosuppression.^4–9^ Genetic or pharmacological depletion of microglia leads to reduction of glioma progression emphasising the importance of tumour–microglia interactions.^5,7,10–12^ Tumour-secreted factors that mediate communication of glioma with microglia and infiltrating myeloid cells could be attractive therapeutic targets in glioblastoma.

Signals for immune cell recruitment in GBM, identities of infiltrating populations and their contribution to tumour progression, are disputable. Macrophage colony stimulating factor (M-CSF, CSF1) and granulocyte macrophages colony stimulating factor (GMCSF, CSF2) are main factors that regulate proliferation and differentiation of myeloid cells.^13,14^ CSF1 signalling was implicated in glioma invasion, as a blockade of CSF-1R affected functions of CD11b^+^ cells (microglia/macrophages), along with glioma invasion in vitro and in vivo.^15,16^ CSF-1R inhibitor BLZ945 reduced tumour growth, without affecting the number of infiltrating CD11b^+^ cells (possibly due to compensation by glioma-secreted CSF2 and interferon γ) but reduced the pro-invasive markers in CD11b^+^ cells.^16,17^ Another CSF-1R inhibitor PLX3397 supressed glioma cell proliferation and tumour growth and interfered with polarisation of immune cells.^18^

CSF2 (granulocyte macrophage colony stimulating factor) stimulates survival, proliferation and differentiation of hematopoietic myeloid cells.^19,20^ Several in vitro studies implicated CSF2 produced by cancer cells in the autocrine regulation of growth of human melanoma, prostate, bladder, gastric colon, skin and non-small-cell lung cancer cells.^21–25^ *CSF2* and its receptor *CSF2R* are co-expressed in human glioma cell lines and GBMs.^26–30^ Secreted CSF2 stimulates glioma cell growth and invasion^26^ but its influence on GBM microenvironment has not been thoroughly explored. We previously reported that Csf2 produced by murine GL261 glioma cells supports microglia-dependent glioma invasion in vitro and tumour growth in mice.^6^

In the present study, we aimed to investigate the role of glioma-secreted CSF2 in controlling glioma–microglia interactions in vitro and in animal models. We demonstrate that depletion of *CSF2* in two human glioma cell lines reduces microglia-dependent glioma invasion in vitro and affects pro-tumorigenic polarisation of microglia. CSF2 knockdown in glioma cells results in impaired recruitment of microglia and macrophages in vivo, reduced glioma growth in mice and improved animal survival.

## Methods

### *CFS2* and immune gene expression in gliomas in the TCGA dataset

Data from five normal brain samples, 248 WHO grade II, 261 WHO grade III and 160 WHO grade IV tumour samples were acquired from TCGA RNAseq repository as data level 3 (FPKM values), quantile normalised and log2 transformed. The *CSF2* expression in normal brain tissues and gliomas of different WHO grades and within molecular subtypes of glioblastoma was compared. Moreover, glioma samples from TCGA dataset were separated into two groups, one with no *CSF2* expression (FPKM = 0) and the other with detectable *CSF2* expression (FPKM > 0.05). Statistical analysis and functional analysis were performed in these two groups (Gene Ontology analysis using clusterProfiler R package).

### Cell cultures

Human glioblastoma cell lines: LN18, LN229, T98G, U251, U87 (ATCC, Manassas, VA) were cultured in DMEM supplemented with 10% foetal bovine serum (FBS, Gibco, MD, USA) and antibiotics (100 U/mL penicillin, 100 μg/mL streptomycin). Jurkat leukaemic T-cell lymphoblast were cultured in RPMI 1640 with 2 mM Glutamine, 10% FBS and antibiotics. Mouse microglia BV2 cell line (ATCC, Manassas, VA) was cultured in DMEM glutaMAX supplemented with 2% FBS and antibiotics. Human immortalised microglia cell line (HM SV40) (Applied Biological Materials Inc.) was cultured in Prigrow III medium (Applied Biological Materials Inc.) supplemented with 10% FBS and antibiotics on extracellular matrix pre-coated flasks. Human astrocytes (Lonza) were grown in Astrocyte Growth Medium (Lonza). Cryopreserved human microglia (1.5 × 10^6^ cells) was purchased (3H Biomedical, Uppsala, Sweden) and grown in Microglia Culture Medium (3H Biomedical) with antibiotics. All cells were cultured in CO_2_/air (5%/95%) at 37 °C (Heraeus, Hanau, Germany).

### Development of stably transfected clones expressing shRNAs

To interfere with the *CSF2* expression two complementary oligonucleotides encoding *CSF2* shRNA with BamH1 and HindIII overhangs were designed: 5′- GATCCAAAGAGAACCTGAAGGACTTTTCAAGAGAAAGTCCTTCAGGTTCTCTTTGTTTTTTGGAAA-3′ and 5′- AGCTTTTCCAAAAAACAAAGAGAACCTGAAGGACTTTCTCTTGAAAAGTCCTTCAGGTTCTCTTTG -3′. The annealed DNA was ligated into the pSilencer 2.0-U6 vector (Ambion, Austin, TX), linearised with BamH1 and HindIII enzymes. The resulting plasmid (shCSF2) was sequenced. pSilencer 2.0-U6 Negative Control (Ambion, Austin, TX) was used as a control (shNeg). U87 and LN18 glioma cells were electroporated with 1.0 µg of plasmid DNA using Amaxa Cell Line Nucleofector Kit (Lonza). The following day, the medium was changed to a complete medium containing hygromycin B (50 µg/ml for U87; 200 µg/ml for LN18). Resistant clones (shCSF2 or shNeg) were selected after 2 weeks and analysed for the expression of *CSF2* mRNA using quantitative PCR (qPCR).

### Quantification of mRNA and protein levels

RNA was isolated using RNeasy kit (Qiagen) and RNA quality/yield was verified using Bioanalyzer 2100 (Agilent Technologies, Santa Clara, CA). Two independent samples of total RNA from non-tumoural human brains pooled from multiple donors were purchased from Ambion and Clontech, and served as control, normal brain samples. The cDNA was synthesised by extension of the oligo(dT)15 primers (2.5 mmol/L) using 200 units of M-MLV reverse transcriptase (Sigma-Aldrich, Germany). The qPCR amplification was performed with SYBR Green PCR mix in the following conditions: 50 °C for 2 min, 95 °C for 10 min, and 40 cycles of 15 s at 95 °C and 1 min at 60 °C using a 7900HT PCR System (Life Technologies). The following primer sequences were used: 5′-ACCTGCCTACAGACCCGCCT-3′ and 5′-GAAGTTTCCGGGGTTGGAGGGC-3′ for human *CSF2*; 5′-CGGACATCTAAGGGCATCAACA-3′ and 5′-AACGAACGAGACTCTGGCATG-3′ for 18S RNA. The relative quantification of gene expression was determined using the comparative CT method. The expression levels were compared with that of 18S RNA. Gene expression in primary cultures of human microglial cells was measured using TaqMan Gene Expression Assays (Life Technologies, Carlsbad, CA). Specific primers and FAM-labelled probe sets for *IL10* (Hs00961622_m1), *MYC* (Hs00153408_m1), *IRF7* (Hs01014809_g1) and *IL1B* (Hs01555410_m1), *GAPDH* (Hs02758991_g1) were used. The reaction consisted of cDNA equivalent to 50 ng RNA, 5 µl Fast TaqMan PCR master mix (Life Technologies, Carlsbad, CA) and 0.5 µl of each primer. Thermal cycling conditions: 10 min at 95 °C and 40 cycles of 95 °C for 15 s and 1 min at 60 °C were employed for annealing and extension.

To determine CSF2 production, human glioma cells and human astrocytes (1 × 10^6^ cells) were seeded on the plates, incubated overnight, then culture conditioned media were collected and analysed by ELISA (Abcam, ab100529).

### Cell survival and proliferation assays

U87 and LN18 glioma cells were cultured in 24-well plates for 24, 48 or 72 h. Cells were incubated with MTT (3-(4,5-dimethylthiazol-2-yl)-2,5-diphenyltetrazolium bromide, Sigma, 0.5 mg/ml) for 3 h and then lysed with a buffer containing 20% SDS and 50% DMF. Cell proliferation was determined using Cell Proliferation ELISA BrdU assay (Roche Diagnostics GmbH, Germany). Briefly, 1.5 × 10^4^ U87 and LN18 glioma cells were seeded onto 96-well plates overnight and then incubated with 10 µM BrdU (5-bromo-2′-deoxyuridine) for 2 h, washed intensively, and fixed. Quantification of incorporated BrdU was performed according to manufacturer’s protocol. Optical densities in MTT or BrdU assays were measured at 570 or 450 nm, respectively, using a scanning multi-well spectrophotometer (Thermo Labsystem Multiscan EX).

### Invasion assay

Invasion assay was performed as described.^5^ Briefly, 24-well cell culture inserts (12 μm pore size Millicell, Millpore, Tullagreen, Ireland) were coated with 1 mg/mL Growth Factor Reduced Matrigel Matrix (BD Biosciences, CA, USA) in DMEM, and dried at 37 °C for 5 h. LN18 and U87 glioma cells were seeded at 2 × 10^4^ on matrigel-covered membrane in an upper compartment and then inserts were transferred to a 24-well plate with or without human SV40 microglial cells (HM SV40), murine primary microglia cultures or BV2 cells in a lower compartment. After 18 h cells invading through the matrigel were fixed with 95% methanol and stained with DAPI (4′,6-Diamidino-2-Phenylindole; 0.01 mg/ml, Sigma). The membranes from inserts were cut out and invading cells were counted using Laser Scanning Cytometry (LSC, CompuCyte). In some experiments, images were acquired using fluorescence microscope (Leica DM4000B, ×10 objective) from five independent fields and numbers of cell nuclei were counted using ImageJ software.

For studies with neutralising anti-CSF2 or anti-CSF2Rα antibodies, mouse BV2 microglial cells or human SV40 microglia were plated onto 24-well plates at the density of 4 × 10^4^. After 24 h, the culture medium was replaced with fresh one containing Ab CSF2 (500 ng/mL, ABCAM, #ab9667), Ab CSFRα (20 ng/mL, PE Mouse Anti-Human CD116, BD Pharmingen™, #551373) or isoAb CSF2R (20 ng/mL, PE Mouse IgG1, κ Isotype Control, BD Pharmingen™, #556650), 1 h before seeding glioma cells onto inserts.

### Cell encapsulation and hollow fibre inoculation

C57BL/6 (8–10 weeks) mice were handled according to governmental (LAGeSo) and internal (Max Delbruck Center, Germany) rules and regulations. Environmental conditions were as follows: temperature of 21 °C ± 2 °C, humidity of 55% ±10%, a standard 12∶12 light:dark cycle. Animals were housed in standard cages and given free access to food and water ad libitum. Mice were anesthetised with i.p. injections of 10 µl of the anaesthetic mixture (containing 0.1% xylazyne and 1.5% ketamine-hydrochloride mixture in 0.9% NaCl_per body weight. The eyes of mice were covered with glycerine fat to avoid cornea drying. Choice of anaesthetics, dosage and way of delivery was according to guidelines of the Local Ethics Committee.

Human LN18 and U87 glioma cells expressing shNeg or shCSF2 (2 × 10^6^ cells/ml) were infused into hollow fibres (HF) (Minntech, Minneapolis, USA). Fibres were cut into 5 mm length pieces and sealed at both ends. Cell number in the fibre was determined and only fibres with an equal number of cells were used. The mouse head was placed onto a stereotactic frame (David Kopf Instruments, Tujunga, USA) under anaesthesia. Through a midline incision, a burr hole was drilled at 1 mm anterior to the bregma and 1.5 mm both right and left side from the midline. Canals were created by inserting a 26-gauge Hamilton syringe 5 mm ventral from dura mater. Then the HFs were inserted into both canals.

After completing the experiment, the animals were anesthetised with an i.p. injection of ketamine (75 mg/kg) and medetomidine (1 mg/kg) and after verification of the unresponsiveness to the noxious stimuli and absent reflexes, mice were transcardially perfused with PBS and 4% paraformaldehyde. Mice were sacrificed and brains were removed and post-fixed for 24 h and placed in 30% sucrose in PBS at 4 °C. Subsequently, the brains were frozen using dry CO_2_ and serial 20-µm-thick coronal sections were collected and stained with toluidine blue.

### Intracranial glioma implantation and quantification of tumour size

The animal study was conducted according to the protocol approved by the Local Ethics Committee (259/2012). Experiments were performed on BALB/c Nude Mouse (CAnN.Cg-*Foxn1*^*nu*^/Crl from Charles River Laboratories, USA), housed in individually ventilated home cages (IVC) in a pathogen free environment. Mice were fed with standard chow ad libitum and kept under standard day/night conditions. Ten weeks old male mice were anesthetised with isoflurane inhalant anaesthesia (4–5% induction, 1–2% maintenance, 21% oxygen) using Isoflurane vaporiser (Temsega, Tabletop Anesthesia Station), and analgesic Butorphanol (1 mg/kg, ip.; Orionvet) and Tolfedine 4% (4 mg/kg s.c.; Vetoquinol) was given as a single injection. Before starting the surgical procedure and during the surgery the depth of anaesthesia was verified by the lack of deep pain response in the limb and breathing regularity. Choice of specific anaesthetics was recommended by the veterinarian and approved by The Local Ethics Committee. After performing a 1 cm longitudinal skin incision at the level of sagittal suture, 2 mm diameter hole was drilled with a micromotor drill (Stoelting) according to the coordinates (1.5 mm AP, 1.5 mm ML). LN18 glioma cells expressing shCSF2 or shNeg (1 × 10^6^ cells in 3 µl of DMEM) were implanted under sterile conditions into the right striatum using 1 ml syringe with a 26-gauge needle in a stereotactic apparatus (Stoelting Co., USA). At day 15th after glioma implantation, the animals were anesthetised with an i.p. injection of ketamine (75 mg/kg) and medetomidine (1 mg/kg) and after verification of the unresponsiveness to the noxious stimuli and absent reflexes, mice were transcardially perfused with PBS and 4% paraformaldehyde . Mice were sacrificed and brains were removed and post-fixed for 24 h and placed in 30% sucrose in PBS at 4 °C. Subsequently, the brains were frozen using dry CO_2_ and serial 20-µm-thick coronal sections were collected and stained with toluidine blue. Images were acquired using a Leica DM4000B microscope. Tumour areas were measured using Leica DM4000B software on every fourth brain slice and tumour volumes were calculated as described.^7^ For survival experiments the weight of the mice with implanted cells was monitored daily and death was recorded.

### Immunofluorescence staining and TUNEL labelling

To detect microglia/macrophages, brain sections were incubated with 10% donkey serum with PBS-0.1% Triton X-100 (PBS-T) for 30 min at room temperature (RT), then stained with a rabbit anti-Iba1 antibody (WAKO, 1:1000) for 24 h at 4 °C followed by incubation with donkey anti-rabbit Alexa Fluor 568 (1:1000, Invitrogen) for 2 h at RT. To visualise astrocytes and microglia, sections were blocked with 5% BSA and 5% donkey serum for 1 h at RT, then stained with the goat anti-Iba1 antibody (Abcam, 1:350) and with the rabbit anti-GFAP (1:1000, Sigma) for 24 h at 4 °C followed by incubation with donkey anti-goat Alexa Fluor 488 (1:1000, Invitrogen) and donkey anti-rabbit Alexa Fluor 555 (1:1000, Invitrogen) for 2 h at RT. Human cells were detected with anti-PCNA antibody (1:500, Sigma). Cell nuclei were counterstained with DAPI (Sigma-Aldrich, 1:1000). Primary antibody was omitted in a negative control. Sections were mounted with Fluorescent Mounting Medium (DAKO) and images were collected using a Leica DM 4000B microscope. Numbers of Iba1^+^ and GFAP^+^ cells were determined using ImageJ software (NIH) in each animal.

For double Iba1+ and TUNEL staining, frozen brain slices were treated with 99% ethanol and 80% acetic acid (2:1) at +4 °C for 30 min. Slices were first incubated for 1 h in a blocking solution: 2% BSA + 1.5% NGS in 0.1% PBS-T, and next with an anti-Iba1 antibody conjugated to Alexa Fluor 647 (diluted 1:1000 Invitrogen) for 2–3 h in RT. Slices were washed three times in PBS and incubated with TUNEL reaction mixture (Roche, In situ cell death detection kit, fluorescein labelled) for 1 h in 37 °C. Incubation was followed by rinsing three times in PBS, dehydration in alcohol and mounting with DPX Mountant for histology (Fluka). Cover slipped brain sections were examined under a fluorescent Olympus IX70 microscope and inverted confocal microscope (DM IRE2, Leica).

### Statistical analysis

All data are presented as mean ± standard deviation (s.d.). Differences between groups were evaluated using one-tailed sample *t*-test (for two groups) and ANOVA (for multi-variant comparisons). In animal experiments *p* value was calculated using the Mann–Whitney *U* test, or Student’s *t*-test using Statistica software (ver. 7.1 StatSoft. Inc, OK, USA) or GraphPad Prism v6.01 (GraphPad Software, Inc., San Diego, CA, USA).

## Results

### *CSF2* is highly expressed in a subset of mesenchymal glioblastomas and cultured human glioma cells

**Fig. 1:**
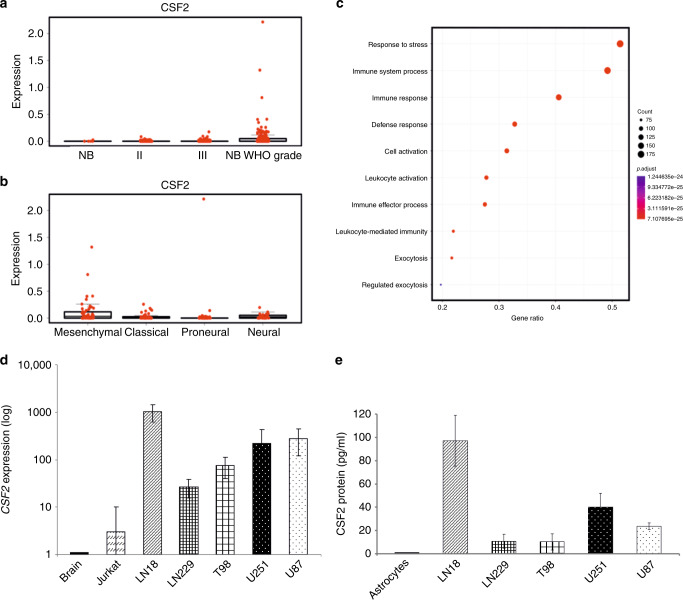
CSF2 expression in gliomas. **a** Boxplots represent CSF2 FPKM values in normal brain samples (NB), low-grade gliomas (WHO grade II and III) and GBM (glioblastoma, WHO grade IV) TCGA datasets. **b** Boxplots represent CSF2 FPKM values in GBM samples with division into molecular subtypes.^31^ **c** Genes overexpressed in CSF2 expressing tumours (FDR *p*-value < 0.05 and FC > 3) were an input for GO enrichment. Dotplot shows the most enriched GO groups in CSF2 expressing GBMs, i.e. related to leukocyte activation, immune and defence responses. **d** Quantification of CSF2 expression using qPCR in healthy brain samples, Jurkat cells and glioma cell lines. **e** CSF2 protein levels in cells culture supernatants from normal human astrocytes and human glioma cell lines were determined using ELISA. Data are presented as means± from three independent experiments, performed in triplicates.

First, we compared the expression of *CSF2* in human gliomas of different WHO grades and glioblastoma subtypes. All data, including the control samples (normal brain samples) were downloaded from the TCGA repository (The Cancer Genome Atlas Research Network, 2008). TCGA contains a limited number of normal brain samples, therefore only five samples were used as a reference to show a basal level of CSF2. The highest *CSF2* expression was found in a subset of patients with GBMs (Fig. 1a). We also checked the distribution of *CSF2* in different molecular subtypes of GBMs defined by the TCGA network.^31^ When compared with other subtypes, *CSF2* was significantly upregulated in the mesenchymal subtype of GBM (Fig. 1b). Furthermore, using computational analysis with GO annotations, we identified functional groups among genes significantly enriched (adjusted *p*-value < 0.01) in gliomas with high *CSF2* expression (FPKM > 0.05) relative to tumour samples with no *CSF2* expression (FPKM = 0). We found over-representation of genes related to immune and stress responses, defence mechanism and leucocyte activation as the most relevant to increased *CSF2* expression (Fig. 1c).

Further, we determined the levels of *CSF2* mRNA using quantitative PCR and its protein by ELISA in five glioblastoma cell lines, normal brain samples and normal human astrocytes (NHA). In comparison to normal brain or NHA, the levels of *CSF2* mRNA (Fig. 1d) were upregulated in human LN18, LN229, T98, U251 and U87 glioma cells. Concordantly, the levels of CSF2 protein measured by ELISA were increased in culture supernatants of all tested human glioma cells (Fig. 1e). LN18 and U87 glioma cells showed the highest levels of *CSF2* mRNA and produced abundant quantities of CSF2 protein, therefore these cells were employed for further experiments.

### Knockdown of *CSF2* in glioma cells reduces microglia-dependent invasion and induces a shift of microglia phenotype

**Fig. 2:**
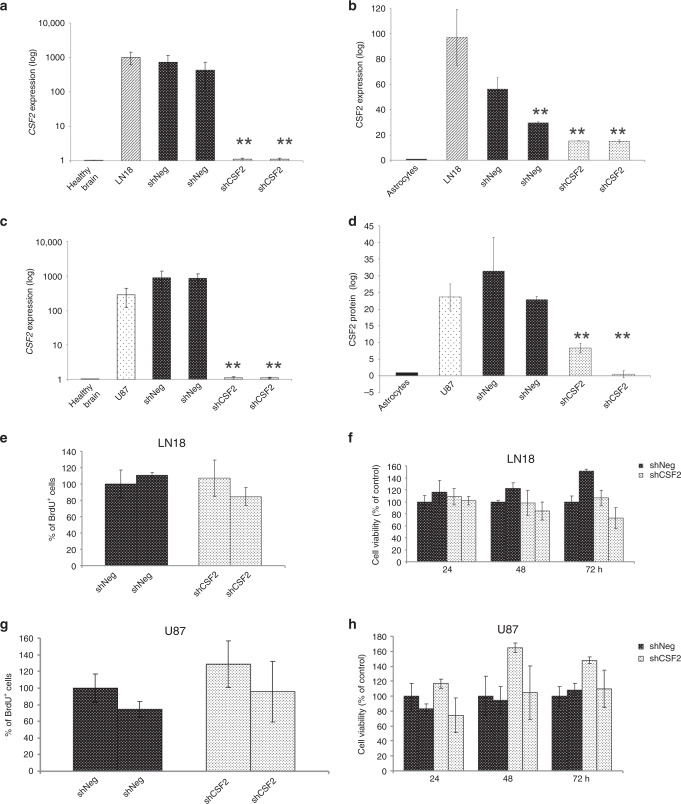
Development and characterisation of CSF2-depleted glioma cells. **a–d** Quantification of CSF2 mRNA (**a**, **c**) and protein (**b**, **d**) levels in parental LN18 glioma cells (**a**, **b**) and U87 glioma cells (**c**, **d**), and two clones of each shNeg and shCSF2 cells; normal human astrocytes and normal brain samples were used as a reference. Bars show means ± s.d., *n* = 3. **e**–**h** CSF2 knockdown did not affect the proliferation (**e**, **g**) and viability (**f**, **h**) of CSF2-depleted LN18 and U87 glioma cells, when compared to control shNeg cells. All results are expressed as the values relative to those obtained for control cells (shNeg1 = 100%) and are presented as the means ± s.d., *n* = 3.

Using RNA interference, we generated LN18 and U87 glioma cells stably depleted of *CSF2* and for each glioma cell line we selected two clones (shCsf2) with the highest reduction of *CSF2* mRNA and CSF2 protein levels, as determined by qPCR (Fig. 2a, c) and ELISA (Fig. 2b, d), respectively. Control cells stably transfected with a non-targeting shRNA (ShNeg) expressed similar levels of *CSF2* as parental cells. Silencing of *CSF2* expression in LN18 and U87 glioma cells did not significantly affect cell proliferation and survival, as demonstrated by BrdU incorporation (Fig. 2e, g) and MTT metabolism tests (Fig. 2f, h), respectively.

**Fig. 3:**
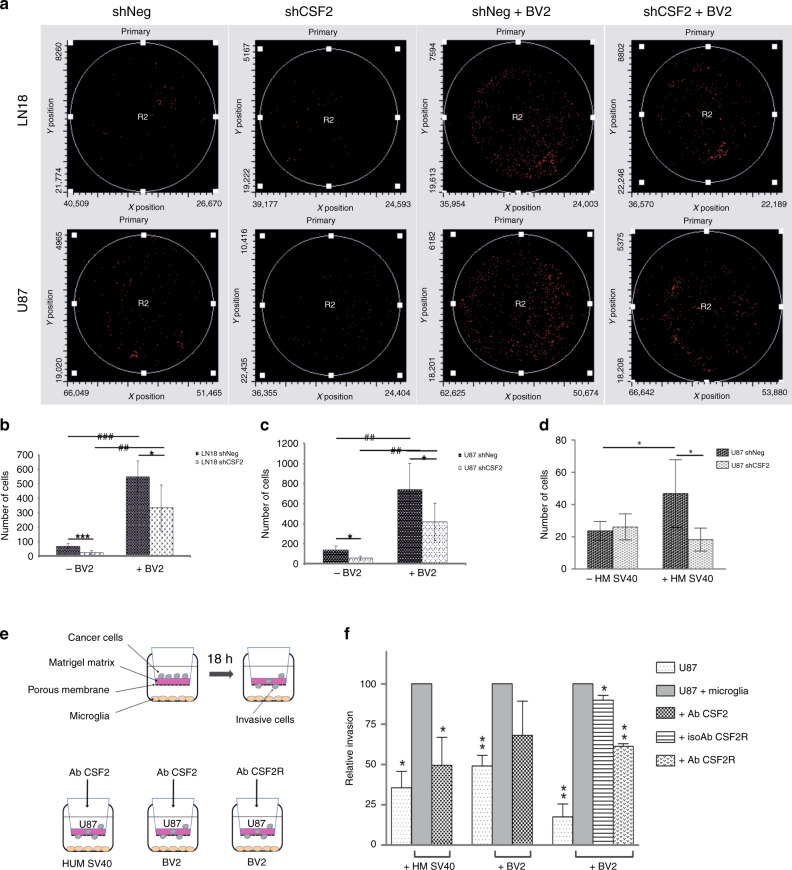
CSF2 knockdown reduces microglia-dependent invasion of human glioblastoma cells. **a–c** Cell invasion was determined using matrigel invasion test. Same number of shNeg or shCSF2 LN18 and U87 glioma cells were plated on matrigel-covered inserts with or without BV2 microglia cells in the lower compartments. Cells migrating through the matrigel were stained and counted with laser scanning cytometry (representative histograms are shown in **a**) or quantified manually in five fields (**b**, **c**). **d** Invasion of shNeg or shCSF2 glioma cells in the absence or presence of human SV40 immortalised microglial cells (HMSV40) was determined. Data are presented as means ± s.d.; statistical significance was analysed by ANOVA and marked as *shNeg versus shCSF2, *p < 0.05; # in the absence versus presence of microglia, #p < 0.05, ##p < 0.01). **e** Schematic representation of tested conditions. **f** The effect of antibodies against CSF2 or CSF2Rα on the invasion of U87 glioma cells co-cultured without or with HMSV40 or BV2 microglia in the lower compartment.

Glioma cells become more invasive upon co-culture with microglial cells.^32^ To study if tumour-derived CSF2 contributes to microglia-dependent invasion, we co-cultured human glioma cells with microglial cells and tested their invasion through a reconstituted basement membrane matrix (Matrigel). Invasion of glioma cells was significantly increased in the presence of both primary microglia cultures and immortalised BV2 microglial cells (Fig. S1), therefore further studies were performed using easily obtainable BV2 microglial cells. Migrating glioma cells were quantified using laser scanning cytometry (Fig. 3a). Knockdown of *CSF2* in LN18 (Fig. 3b) and U87 (Fig. 3c) glioma cells strongly reduced BV2-dependent invasion as compared to shNeg controls. Similar effects were observed in the co-culture with immortalised human SV40 microglia (HM SV40), i.e. HM SV40-stimulated invasion was decreased in *CSF2*-depleted glioma cultures (Fig. 3d). Moreover, to confirm the role of CSF2 signalling in microglia-dependent glioma invasion, we employed neutralising antibodies against CSF2 or its receptor CSF2Rα (Fig. 3e). Anti-CSF2 antibody significantly reduced the stimulating activity of human SV40 microglial cells on glioma invasion and to a lesser extent interfered with BV2 cells—dependent effects (Fig. 3f). Concordantly, the antibody against CSF2Rα decreased the invasiveness of glioma cells, which was induced by the co-culture with BV2 cells; an isotype control antibody had a negligible effect.

Tumour-infiltrating microglia and macrophages display the pro-tumorigenic, immunosuppressive phenotype in gliomas.^1^ We studied the expression of genes recognised as markers of pro-tumorigenic (*IL10, MYC*) and inflammatory (*IRF7*, *IL1B*) phenotypes in primary human microglia cultures treated with LN18 and U87 glioma conditioned medium (GCM). GCM from control LN18 and U87 cells (shNeg) induced *IL10* and *MYC* expression in human microglia, while knockdown of *CSF2* in glioma cells reverted *IL10* mRNA to the basal levels. Moreover, we observed upregulation of *IRF7* mRNA and *Il1b* mRNA in human microglia upon stimulation with *CSF2*-depleted LN18 or U87 glioma cells, respectively, relative to treatment with shNeg-conditioned medium (Fig. S2). This indicates a shift of microglia from anti- to pro-inflammatory phenotype in the absence of tumour-derived CSF2.

### Tumour-derived CSF2 controls recruitment of microglia and macrophages and contributes to tumour progression

**Fig. 4:**
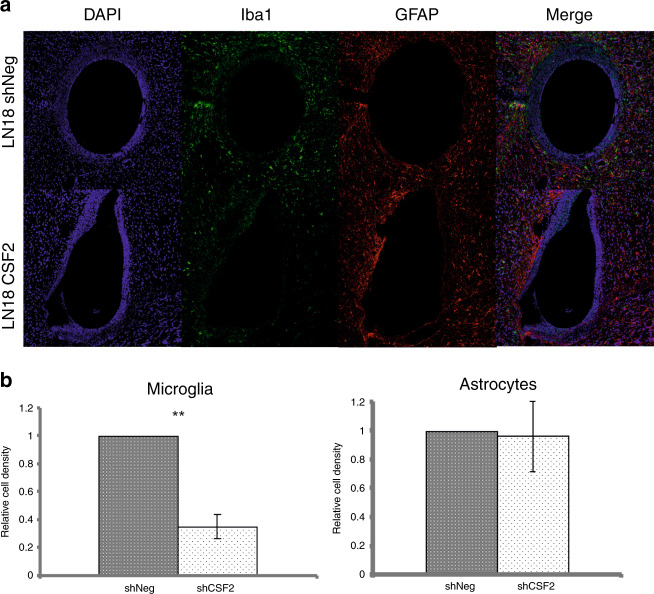
Recruitment of microglia, but not astrocytes, is impaired in CSF2-depleted gliomas. **a** CSF2 depleted or shNeg LN18 glioma cells encapsulated in hollow fibres were transplanted to hemispheres of murine brain. After 14 days, mice were sacrificed and brain sections were stained by immunofluorescence using anti-Iba1 (green) and anti-GFAP (red) antibodies. Cell nuclei were counterstained with DAPI (blue). **b** Quantification of Iba1+ cells (microglia/macrophages) and GFAP+ cells (astrocytes) was performed by counting positive cells in 5 random fields. Data is presented as a relative cell density versus the hemisphere with shNeg-containing hollow fiber. The statistical significance was determined using Student’s t test, *p<0.05, **p<0.01; (mean ± s.d., n = 4 mice).

To study the impact of tumour-derived CSF2 on the brain microenvironment, we encapsulated human U87 and LN18 glioma cells into hollow fibres (HF) and transplanted them into the brains of immunocompetent mice. Hollow fibres were permeable to tumour-derived soluble factors, but prevented direct interactions between tumour cells and microenvironment, and thus precluded the immune system activity towards foreign cells.^33^ HFs filled with control (shNeg) and CSF2-depleted (shCSF2) glioma cells were implanted into either hemisphere of the same mouse brain. After 2 weeks, the histological analyses of the implants showed that the outer membranes of the shNeg LN18 and shNeg U87 fibres were surrounded by Iba1^+^ cells, while significantly less cells migrated towards the HF with CSF2-depleted LN18 and U87 glioma cells. Density of Iba1^+^ cells in the areas surrounding the encapsulated CSF2-depleted LN18 or U87 gliomas was, respectively, 2.8-fold (Fig. 4a) or 1.4-fold (Fig. S3) lower than around the shNeg-containing HF. There was no change in infiltrating GFAP-positive astrocytes surrounding glioma-filled fibres (Fig. 4 and Fig. S3).

**Fig. 5:**
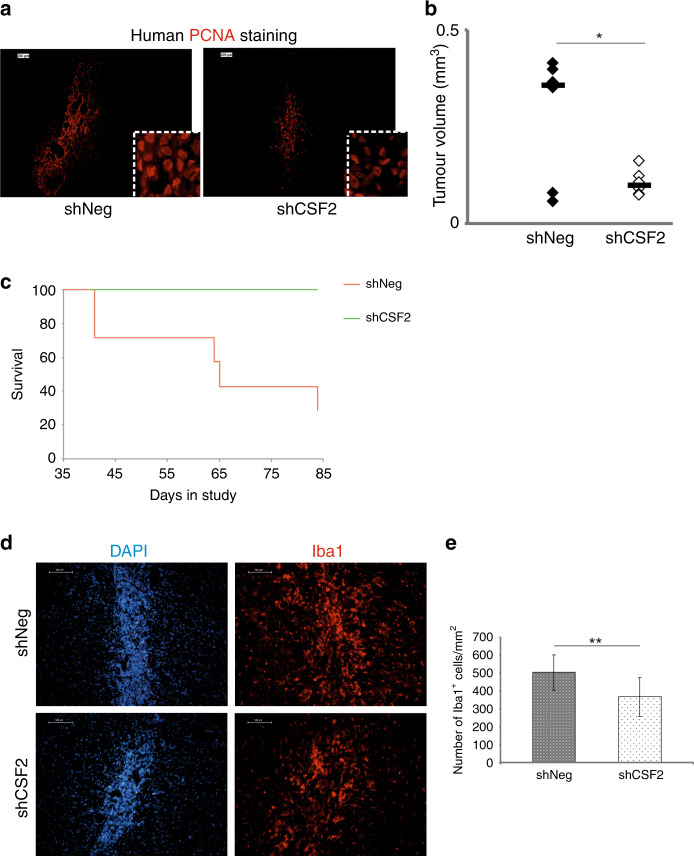
Myeloid infiltrates and tumour growth in control and CSF2-depleted gliomas. **a** ShNeg or shCSF2 LN18 glioma cells were implanted into the striata of athymic mice and the tumours kept growing for 15 days. Human cells were visualized in brain sections with anti-human PCNA staining; scale bar 100 µm. Inset shows cells at higher magnification. **b** Quantification of tumour volume of shNeg or CSF2-depleted gliomas. Each dot represents an individual animal, and the bold line depicts the mean (8 mice/shCSF2 group, 6 mice/shNeg group); statistical analysis with U Mann-Whitney test; p = 0.01. **c** Survival of mice with implanted shCSF2 gliomas (green) compared with mice with control gliomas (red) (p = 0.04). **d** Microglia/macrophages were visualized using Iba1 staining (red); cell nuclei were counterstained with DAPI (blue). e Quantification of Iba1+ cells in experimental gliomas (8 mice/shCSF2 group, 6 mice/shNeg group). The statistical significance was determined using Student’s t test, *p<0.05, **p<0.01, ***p<0.001.

Next, control (shNeg) and CSF2-depleted (shCSF2) LN18 glioma cells were intra-cranially implanted into striata of athymic mice. Human cells were detected by staining for human PCNA (proliferating cell nuclear antigen). Growth of shCSF2 tumours was significantly reduced relative to controls (shNeg), as shown by anti-PCNA immunofluorescence (Fig. 5a) and histologic evaluation of the tumour volume (Fig. 5b). Survival of mice with CSF2-depleted gliomas was considerably prolonged (Fig. 5c). Intra-tumour infiltration of microglia and macrophages, evaluated with Iba1 staining, was decreased in CSF2-depleted LN18 gliomas compared to control shNeg tumours (Fig. 5d, e).

**Fig. 6:**
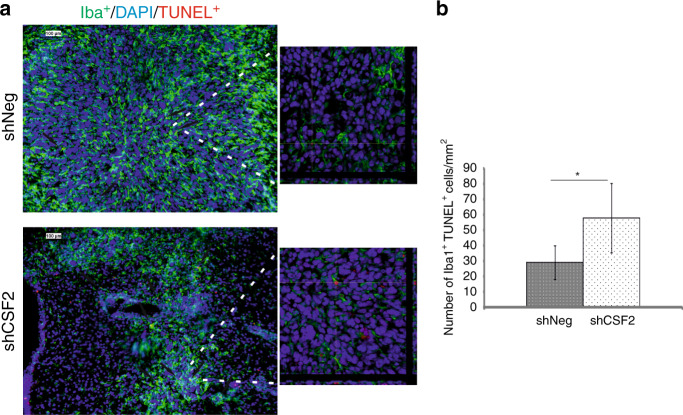
Apoptotic microglia/macrophages in CSF2-depleted gliomas. **a** Microglia/macrophages were visualized using Iba1 staining (green); cell nuclei were counterstained with DAPI (blue). DNA fragmentation was detected by TUNEL staining. Scale bar 100 µm. A right panel shows confocal microscopy images with z-stack projections and orthogonal views along the x and y axes, which demonstrate TUNEL+ nuclei in Iba1+ cells. **b** Quantification of TUNEL+Iba1+ cells in experimental gliomas (8 mice/shCSF2 group, 6 mice/shNeg group). The statistical significance was determined using Student’s t test, *p<0.05, **p<0.01.

CSF1 and its receptor CSF-1R support proliferation and survival of mononuclear phagocytes through the DAP12-β-catenin axis, which controls the expression of survival genes.^34^ We explored if tumour-derived CSF2 is important for survival of infiltrating microglia and macrophages by double staining of the glioma-bearing brain sections for Iba1 and TUNEL (Terminal dUTP nick-end labelling of DNA) that allows to identify DNA fragmentation and stains apoptotic cells. Confocal microscopy analysis revealed significant increase of the number of Iba1^+^ TUNEL^+^ cells in shCSF2 gliomas as compared to shNeg tumours (Fig. 6a, b). This demonstrates that glioma-derived CSF2 is important in supporting both accumulation and survival of microglia and macrophages.

## Discussion

Tumour-infiltrating microglia and macrophages support glioma progression via multiple mechanisms and make a plausible target in designing new therapeutic strategies.^5,7,11,12^ Studies with CSF-1R inhibitors in transgenic or experimental murine gliomas point to a role of CSF1 signalling in mediating glioma–microenvironment interactions.^16,17^ However, *CSF1* mRNA levels were not elevated in GBMs in comparison to normal brains or benign astrocytomas.^6^ Moreover, we found that *CSF2* mRNA is elevated in a subset of GBMs, particularly in the mesenchymal GBM subtype being the most aggressive. Correspondingly, GBMs with high *CSF2* mRNA levels have upregulated expression of immune/wound healing genes, which indicates extensive microglia and macrophage infiltration.

We demonstrate that CSF2/GM-CSF (produced by human LN18 and U87 glioma cells) stimulates microglia to enhance tumour invasion. *CSF2* knockdown in glioma cells or interference with CSF2 signalling using anti-CSF2 or anti-CSF2Rα antibodies significantly reduced microglia-dependent invasion in vitro. CSF2 secreted by glioma cells stimulated microglia accumulation around of LN18 and U87 gliomas encapsulated in hollow fibres in vivo. CSF*2*-depleted LN18 gliomas, when growing in nude mice, were less infiltrated by Iba^+^ cells and formed smaller tumours as compared to gliomas derived from shNeg cells. Moreover, mice harbouring shCSF2 gliomas lived longer than those with control tumours. Interestingly, knockdown of *CSF2* in glioma cells did not affect astrocyte activation and accumulation.

In fact, both glioma cells and immune cells are able to produce and respond to CSF2/GMCSF.^26,27,35,36^ This cytokine was shown to increase glioma migration and protect certain glioma cells from apoptosis.^26,27^ CSF2/GMCSF may stimulate production of other growth factors (nerve growth factor, granulocyte colony stimulating factor, vascular endothelial growth factor). Receptors for those cytokines are expressed on glioma cells, endothelial and stromal cells, and their interplay may contribute to formation of new blood vessels.^35,36^ We found fragmented DNA in some Iba1^+^ cells in shCSF2 gliomas, indicating ongoing cell death. This observation may indicate a supportive role of granulocyte macrophage colony stimulating factor for survival of microglia and macrophages in the tumour microenvironment. CSF1/M-CSF signalling maintains proliferation and survival of monocytes through DAP12-β-catenin signalling pathway,^34^ and CSF2/GMCSF has been shown to regulate β-catenin signalling during myeloid lineage differentiation,^37^ and contribute to the survival of infiltrating monocytes in rat gliomas.^38^

We show that LN18 and U87 glioma polarise primary human microglia cultures towards the pro-tumorigenic, immunosuppressive phenotype (up-regulation of *IL10*, MYC) in vitro. *CSF2* depletion from both LN18 and U87 glioma cells blocked up-regulation of the anti-inflammatory *IL10* expression and led to induction of *IRF7* and *IL1β* mRNA levels in stimulated microglia. This may indicate a shift towards a pro-tumorigenic phenotype upon glioma cells-derived CSF2. However, due to a small set of tested genes, at this stage it is an assumption. Previous studies demonstrated that in vitro CSF2-treated monocytes differentiate into inflammatory macrophages, whereas CSF1-treated monocytes acquire an anti-inflammatory phenotype.^39–41^ This distinct response is attributed to different mediators: the interferon regulatory factor (IRF) 5 is an inducer of inflammation-related genes,^42^ whereas IRF4 and Klf4 control anti-inflammatory macrophage polarisation genes.^43,44^ Interestingly, in human monocytes, IRF4 is more robustly upregulated than IRF5 after CSF2 addition.^45^ The response to CSF2 in the tumour microenvironment is more complex and may be affected by other cytokines or tumour-derived metabolites. For example, in the presence of lactate, which is abundant in the tumour milieu as of Warburg effect, CSF2-activated macrophages cannot produce pro-inflammatory cytokines, but rather, generate vast quantities of anti-inflammatory cytokines.^46^ Extensive use of CSF2 as an adjuvant to anti-cancer vaccines may be thus controversial, unless suited to cancer-specific conditions.^47^

In GBMs, up-regulated CSF2 production, as indicated herein and in previous studies, promotes immunosuppressive activity of tumour-infiltrating myeloid cells.^38,48^ Moreover, CSF2 contributes to systemic immune deficits due to generation of neutrophilia and lymphopenia in GBM patients.^48,49^ Immunosuppressive myeloid cells in glioma microenvironment constitute a barrier to emerging immunotherapies. Recent single-cell RNAseq data, including ours, suggest that this immunosuppressive population in both experimental and human gliomas originates both from blood-derived monocytes and brain-resident microglia.^50,51^ Monocytes, recruited into the tumour by chemotaxis, upregulate the expression of immunosuppressive cytokines and carry on programmed death ligand 1 (PD-L1), a suppressor of an adaptive immune system.^50,51^ It would be of interest to analyse effectiveness of the combined anti-GBM treatment using immune checkpoint PD-1/PD-L1 blockade with alleviation of CSF2-mediated recruitment and immunosuppressive polarisation of myeloid cells.

Taken together, our results point to an important role of tumour-derived CSF2 in the pathology of human gliomas. We demonstrated the up-regulated expression of *CSF2* in TCGA data set herein and in GBM biopsies.^6^ The role of CSF2 in the tumour-driven accumulation of microglia and macrophages and its contribution to glioma progression and immunosuppression suggest that CSF2 signalling might be a novel target for glioblastoma therapy. The results could pave a way to new modalities, in which targeting myeloid cells is combined with immune check-point blockade immunotherapies.

## Supplementary information

Supplemental material

## Data Availability

All data presented within the article and its supplementary information files are available upon request from the corresponding author.
