# Supplementary material for: Tumour-derived CSF2/granulocyte macrophage colony stimulating factor controls myeloid cell accumulation and progression of gliomas
Source: Br J Cancer. 2020 May 11;123(3):438–48. doi: 10.1038/s41416-020-0862-2 (PMC7403321; doi:10.1038/s41416-020-0862-2)
Supplement: Supplementary file 1 — Supplemental material [file 41416_2020_862_MOESM1_ESM.pdf]

Supplementary figures

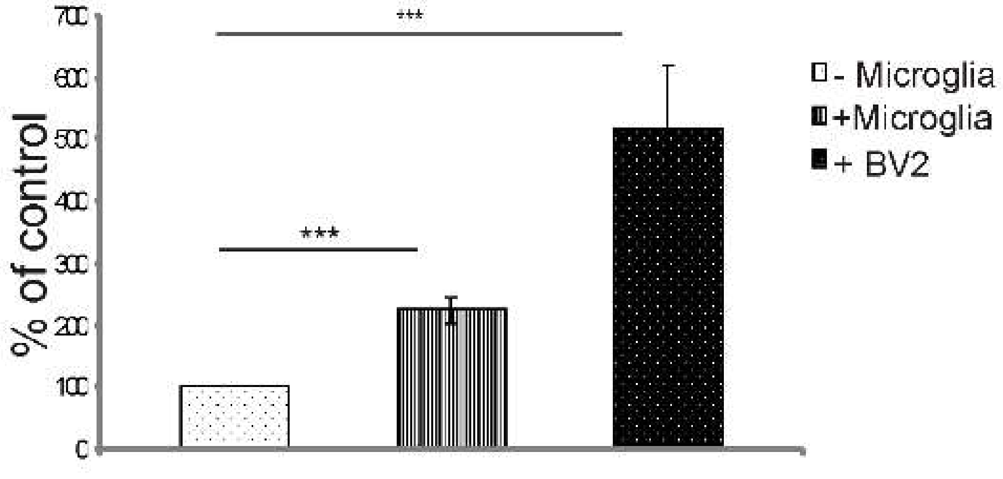

Figure S1. Microglia-dependent invasion of human LN18 glioblastoma cells. Glioma invasion was determined without or with primary microglia or BV2 co-cultures. Data are presented as means ± s.d.; statistical significance was analysed by ANOVA, ***$p<0.001$, n=3, in triplicates.

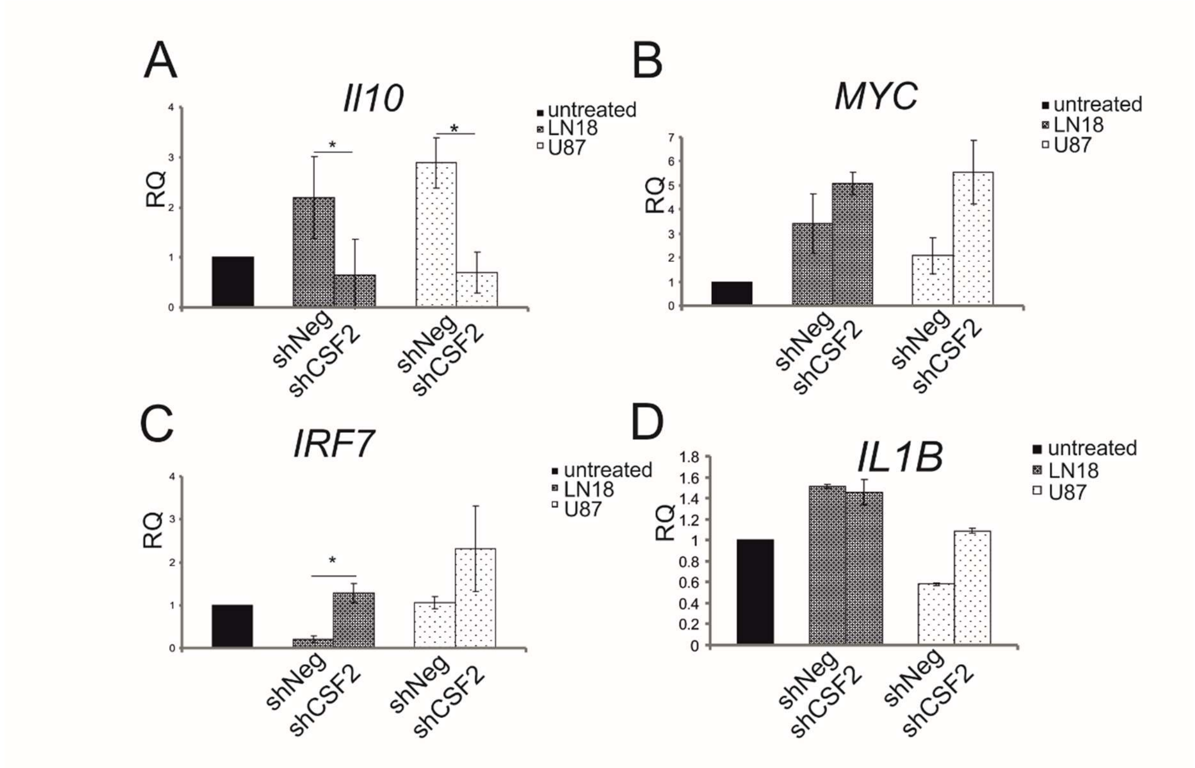

Figure S2. Glioma cells polarize human microglia towards the immunosuppressive phenotype**.** The relative expression of *IL10, MYC* (**A,B**) and *IRF7*, *IL1B* (**C,D**) genes was determined using qPCR in primary human microglia cultures treated with shNeg and shCSF2 glioma-conditioned media (GCM) (means ± s.d.; 2 independent experiments, in triplicates; *p<0.05).

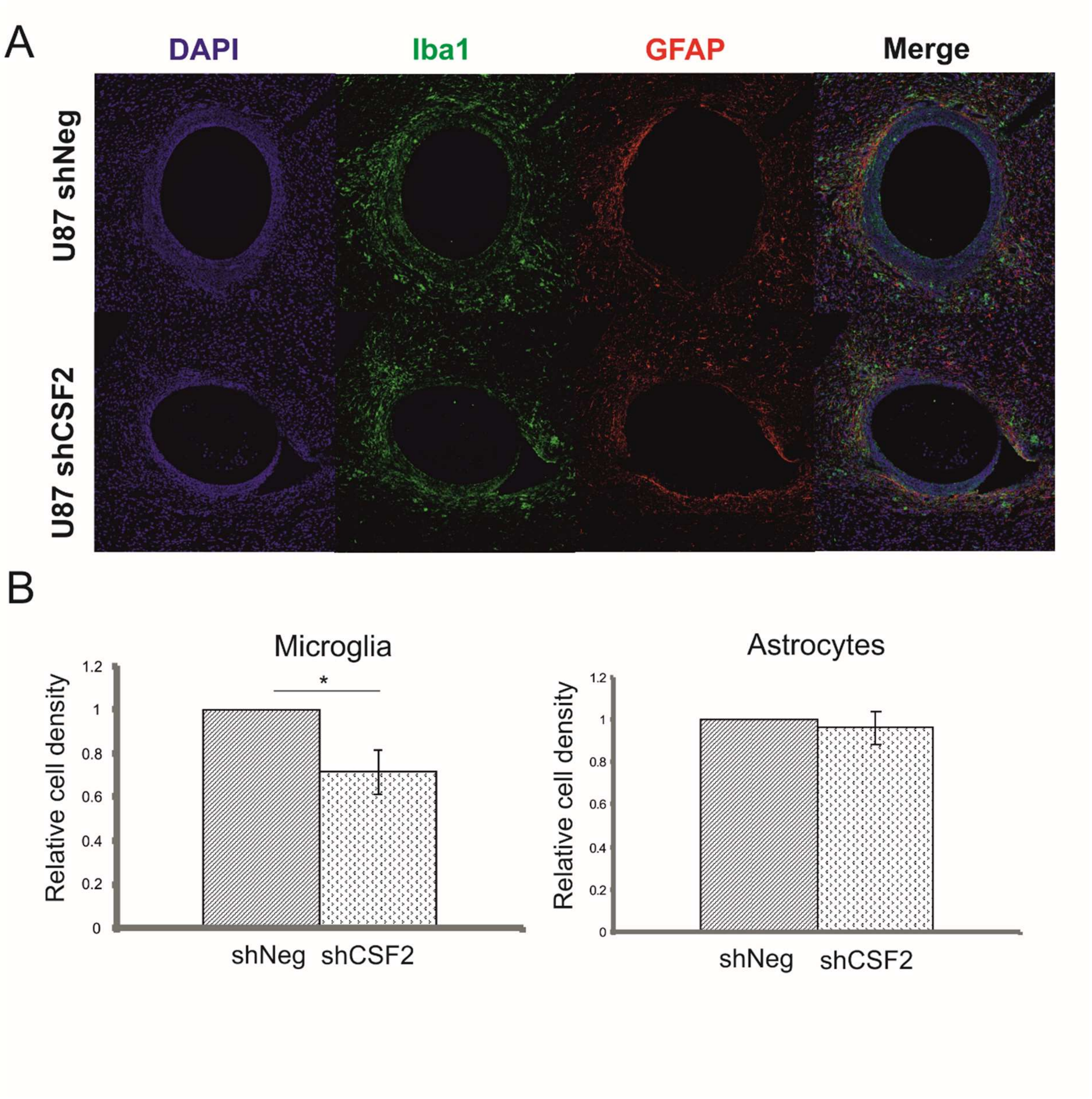

Figure S3. Microglia/macrophage recruitment in CSF2-depleted U87 gliomas. **A** Brain sections with shNeg and CSF2 depleted U87 gliomas (shCSF) encapsulated into hollow fibres were stained with anti-Iba1 (green) and anti-GFAP (red). **B** Quantification of Iba1^{+} and GFAP^{+} cells was performed as described.
